# Congenital tunnel‐shaped ventricular septal defect

**DOI:** 10.1002/ccr3.8017

**Published:** 2023-12-05

**Authors:** Mohammad Reza Eftekhari, Roya Parkhideh, Meysam Khoshavi, Akram Nakhaee

**Affiliations:** ^1^ Department of Cardiology, Imam Khomeini Hospital Complex Tehran University of Medical Sciences Tehran Iran

**Keywords:** adult congenital heart disease, congenital heart disease, tunnel shaped VSD, ventricular septal defect

## Abstract

We report a 47‐year‐old man who presented with right‐sided heart failure. Transthoracic echocardiography revealed a tunnel‐shaped communication (ventricular septal defect) between the left ventricle and the right ventricle with a significant left‐to‐right shunt. The VSD is connected to the lateral wall of the right ventricle by a large tunnel.

## Graphical abstract

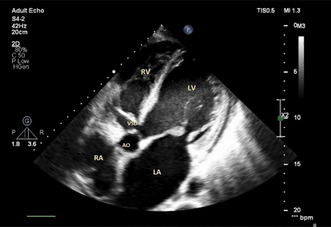

A 47‐year‐old man was admitted to the emergency department (ED) with lower limb edema, ascites, and dyspnea. The patient had a history of ischemic cerebrovascular accident (CVA) and deep vein thrombosis 1 year before the current admission. No history of any cardiac surgery was reported by the patient. Initially, right‐sided heart failure was diagnosed in the patient. He was afebrile and normotensive, and oxygen saturation (SpO_2_) was 90% in room air.

Clinical examination revealed tachycardia, tachypnea, marked jugular venous distension, a high‐frequency holosystolic murmur in the left parasternal border at the fourth intercostal space, ascites, hepatomegaly, and lower limbs pitting edema.

Electrocardiogram (ECG) findings were atrial fibrillation with a rapid ventricular response, significant electrical alternans, and low voltages in limb leads.

Chest computed tomography (CT) scan demonstrated cardiomegaly, bilateral pleural effusion, and evidence of pulmonary venous congestion.

Initial transthoracic echocardiography revealed severe LV enlargement with moderate LV systolic dysfunction (LVEF: 35%–40%), mild LVH, severe right ventricular enlargement with severe systolic dysfunction, severe biatrial enlargement, flail AMVL with severe mitral regurgitation, torrential tricuspid regurgitation, and mild‐ to moderate‐pulmonary and aortic regurgitation.

During the echocardiography, an abnormal turbulent systolic flow directed to the lateral wall of the right ventricle from the lateral side of the tricuspid valve annulus drew our attention. By the clockwise tilting of the probe, a strange finding was detected. There was a defect in the perimembranous part of the interventricular septum which is directed to the lateral wall of RV by a large complete tunnel with a significant left‐to‐right shunt (PPG = 56 mmHg). The VSD flow was entered to RV just near the lateral wall (instead of the septal side). The length of the tunnel was about 40 mm, but the diameter of its exit from the left ventricle was about 9.5 mm and the diameter of the entrance to the right ventricle was about 6.5 mm. Transesophageal echocardiography was done and confirmed TTE findings (Figures 1, 2, 3, 4; Videos [Link], [Link]).

**FIGURE 1:**
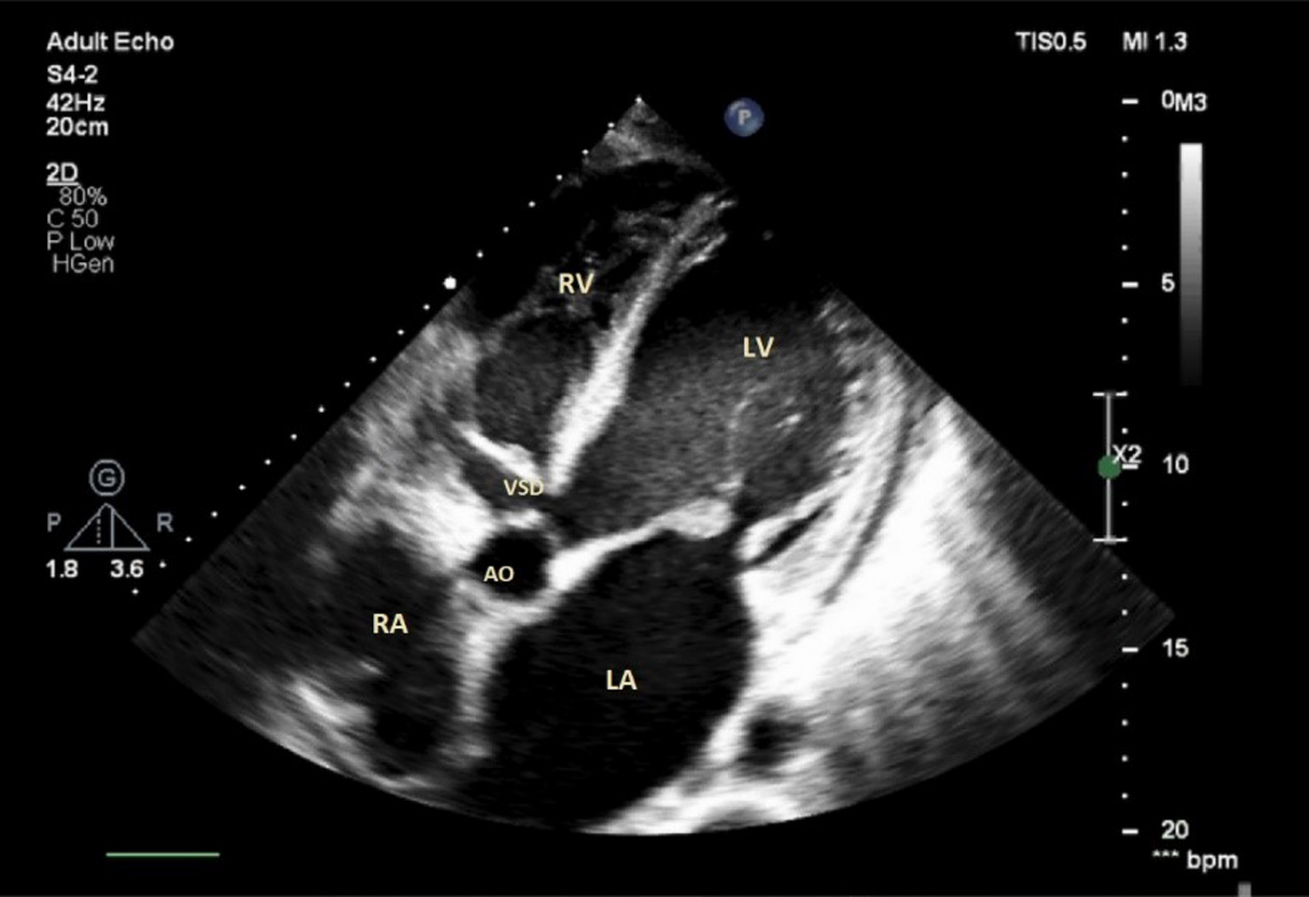
Transthoracic echocardiography in off‐axis view shows a tunnel shape VSD. LA, left atrium; LV, left ventricle; RA, right atrium; RV, right ventricle; VSD, ventricular septal defect.

**FIGURE 2:**
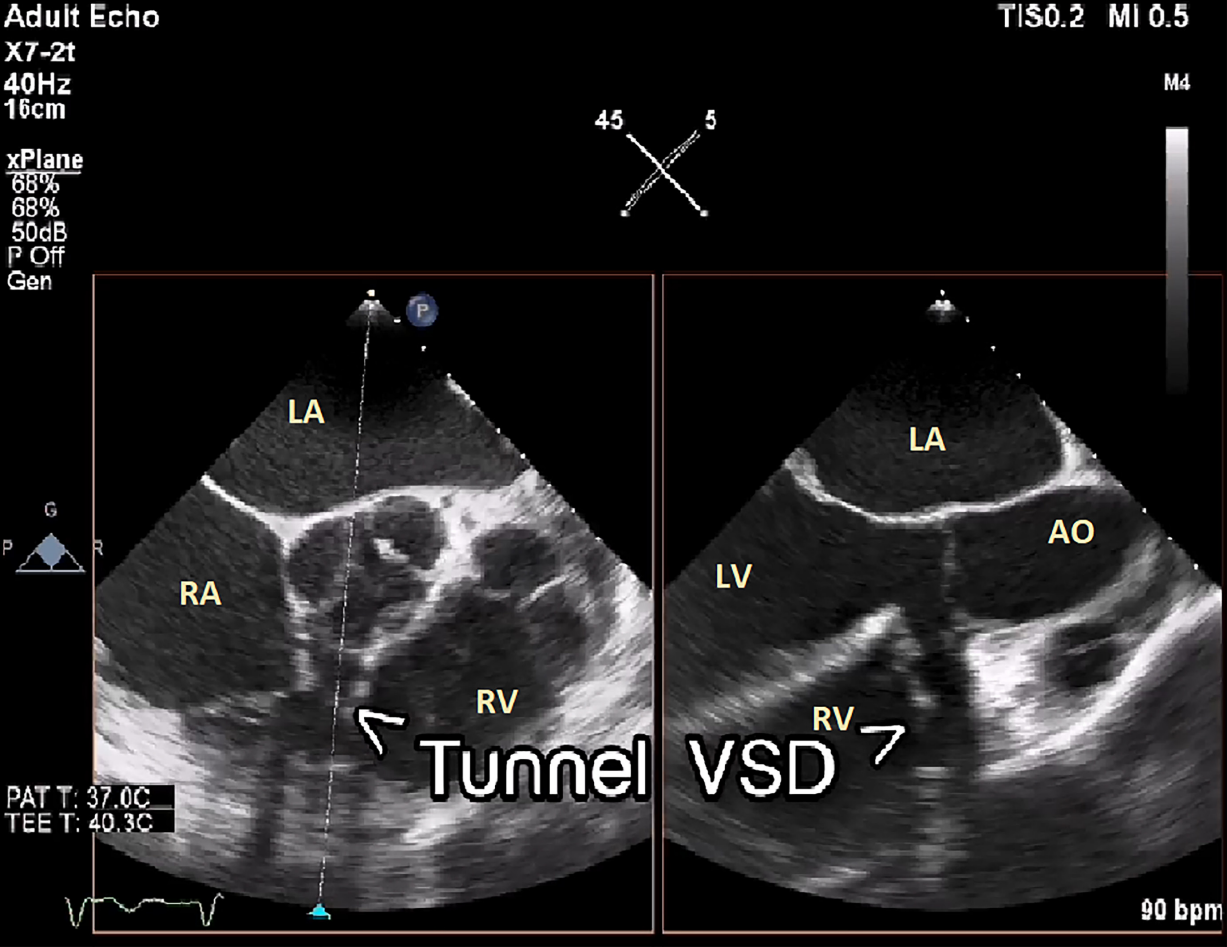
Transesophageal echocardiography at midesophageal level using the biplane method shows a tunnel shape VSD. AO, ascending aorta; LA, left atrium; LV, left ventricle; RA, right atrium; RV, right ventricle; VSD, ventricular septal defect.

**FIGURE 3:**
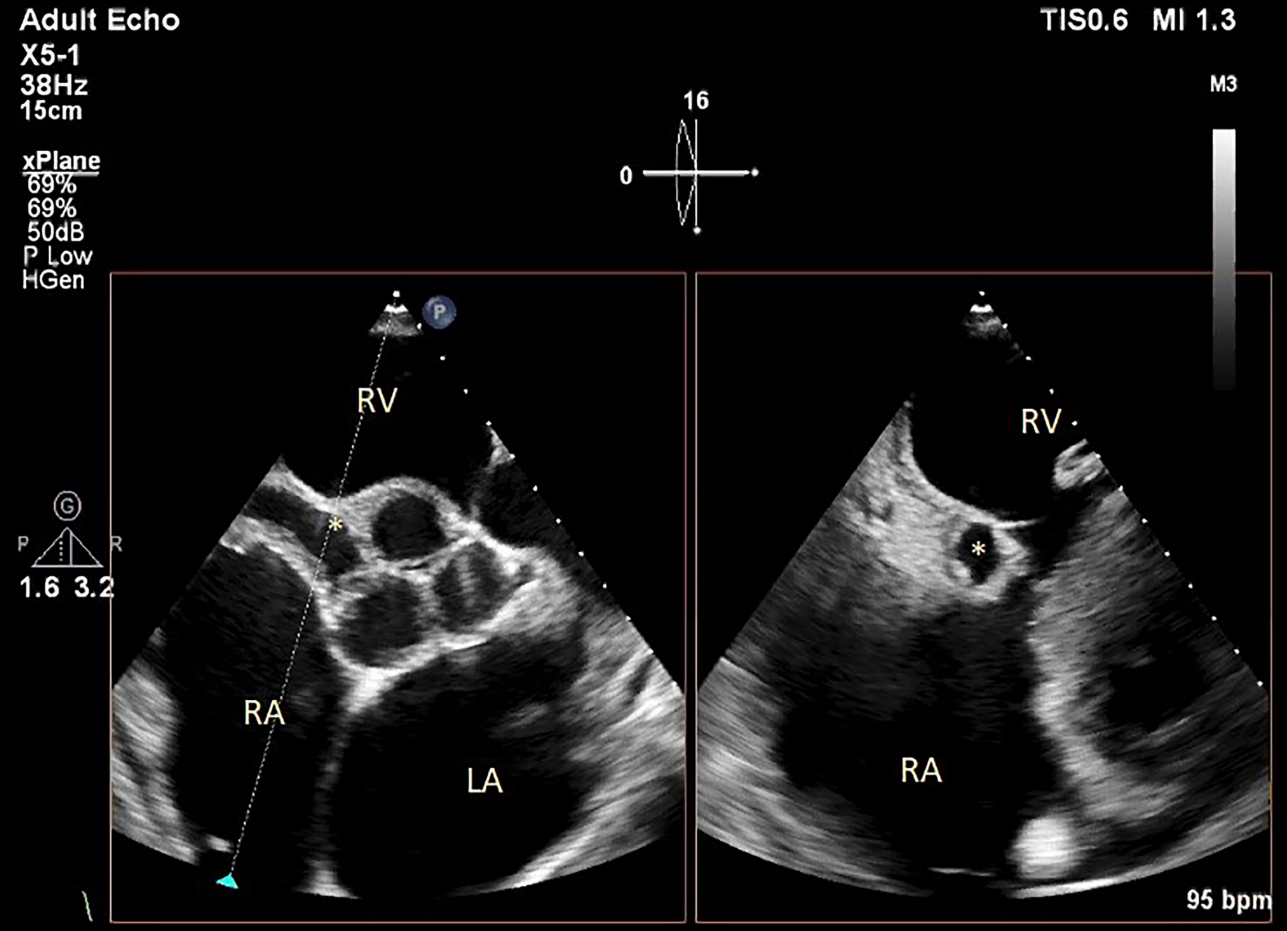
Transthoracic echocardiography using the biplane method shows a tunnel shape VSD (*). LA: left atrium; RA: right atrium; RV: right ventricle.

**FIGURE 4:**
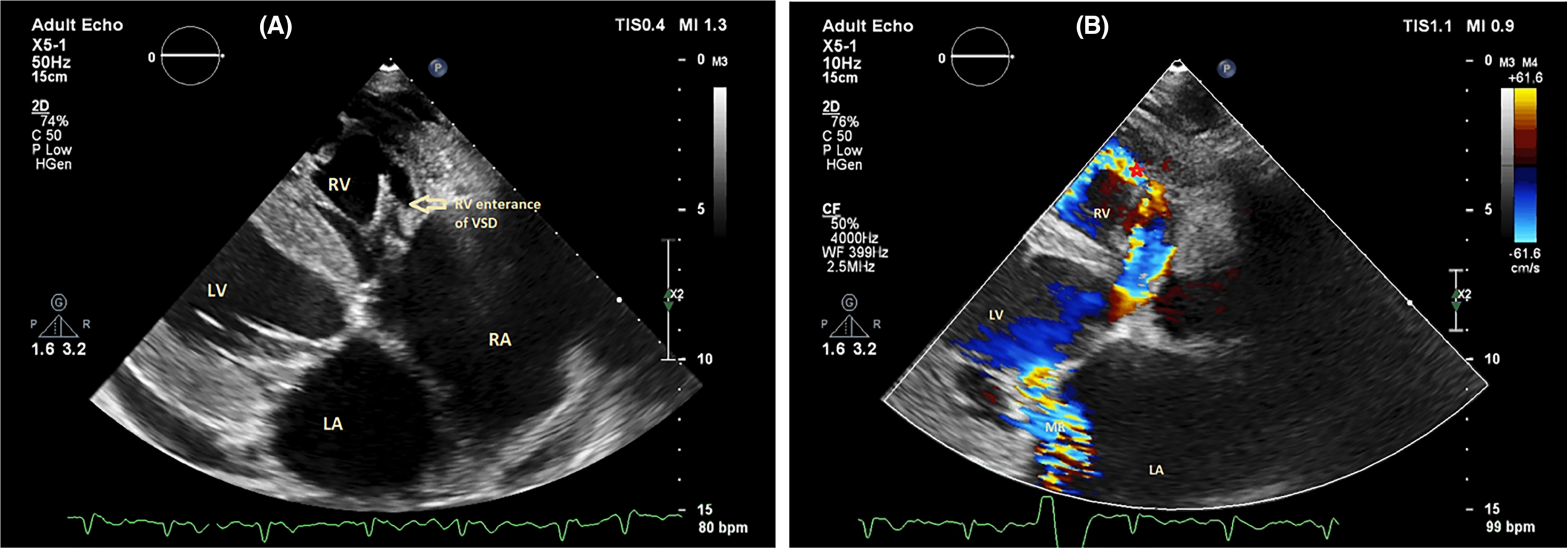
Transthoracic echocardiography in the off‐axis view shows the RV entrance site of VSD tunnel (A), Color Doppler image shows a turbulent flow of VSD. LA, left atrium; LV, left ventricle; MR, mitral regurgitation; RA, right atrium; RV, right ventricle; VSD, ventricular septal defect.

Full heart failure treatment by guideline‐directed medical therapy (GDMT) was done including B‐blocker, diuretics, angiotensin‐converting enzyme (ACE) inhibitors, and mineralocorticoid receptor antagonists. Consequently, the patient underwent right heart catheterization. It was confirmed that a significant shunt from the left to right ventricle exists.

## 1 DISCUSSION

Ventricular septal defect (VSD) or interventricular communication is the most common congenital heart defect.^1^ VSD was described by the International Society for Nomenclature of Pediatric and Congenital Heart Disease as a congenital cardiac abnormality in which there is a hole or pathway between the ventricular cavities.^1^ VSDs are classified as acquired or congenital.^2^

By the end of fourth week of gestation, the bulboventricular cavity formed after the conus cordis and proximal part of the bulbus cordis have merged into a primitive ventricle.^3^, ^4^ In the developing process of formation of the interventricular septum, three main events happen as follows: (1) proliferation of the right balloon ridge near the tricuspid orifice, (2) the proliferation of the left balloon ridge near the mitral orifice, and (3) the proliferation of posterior (atrioventricular) endocardia cushion.^3^, ^4^ Anderson H and colleagues expressed that the membranous portion of the interventricular septum was closed by fibrous continuity between the aortic root and tricuspid valvar leaflets.^4^ VSD may be caused mostly by one of these malformations: deficient development of proximal conus, failure of the fusion of muscular portion to conus septum, and failure of endocardial cushions to fuse.^5^ In the latest classification, the congenital type of VSD is sub‐categorized as perimembranous, juxta‐arterial or doubly committed and muscular based on their geographical location.^1^

The defining feature of the reported patient was the presence of a pathway within the cavity of the right ventricle that coursed blood flow from the left ventricle into the lateral side of the right ventricular cavity by a channel. According to the location of the VSD, it seems that failure and maldevelopment of fibrous continuity between the aortic root and tricuspid valve is the main reason for the tunnel formation of VSD.^4^

Several previous studies have revealed that muscular and perimembranous VSDs could be closed spontaneously depending on the site of the defect. It has been hypothesized that an aneurysm of the membranous septum and leaflet of the tricuspid valve was the most responsible for the closure in perimembranous VSD.^6^, ^7^ Although VSD in the mentioned patient was not spontaneously closed, it seems that the membranous portion of the interventricular septum had abnormally elongated up to RV‐free wall with a defect at that site resulting in this abnormality (VSD).

Although large‐sized VSDs are highly symptomatic at early childhood and should be treated promptly to prevent Eisenmenger, approximately 90% of isolated small VSDs close spontaneously at an early age or rarely remain asymptomatic until older age.^8^ In our case, late presentation could be due to small LV and RV orifices of defect. On the other hand, the amount and speed of blood flow is limited due to the friction of the tunnel wall, and this causes the amount of shunt to decrease.

Our case is noteworthy because the patient did not have any history of cardiac surgery and this type of congenital tunnel shaped VSD has not been reported in the literature.

## AUTHOR CONTRIBUTIONS

**Mohammad Reza Eftekhari:** Methodology; project administration; resources; writing – original draft; writing – review and editing. **Roya Parkhideh:** Writing – original draft; writing – review and editing. **Meysam Khoshavi:** Resources; writing – review and editing. **Akram Nakhaee:** Project administration; resources; writing – original draft; writing – review and editing.

## FUNDING INFORMATION

The authors have no relevant financial or non‐financial interests to disclose.

## CONFLICT OF INTEREST STATEMENT

The authors have no conflicts of interest to declare relevant to this article's content.

## CONSENT

Written informed consent was obtained from the patient to publish this report in accordance with the journal's patient consent policy.

## Supporting information


Video S1.

Video S2.

Video S3.

Video S4.

Video S5.

## Data Availability

All data are available.
